# Dental Students’ Knowledge and Opinion on Optimizing Antibiotic Prophylaxis Guidelines: A Cross-Sectional Survey in India

**DOI:** 10.7759/cureus.115959

**Published:** 2026-09-08

**Authors:** Vaibhavi S Patane, Ashwinirani SR, Girish Suragimath, Madhura Mahajan, Nomaan A Shamlik, Trupti S Patane

**Affiliations:** 1 Oral Medicine and Radiology, School of Dental Sciences, Krishna Vishwa Vidyapeeth (Deemed to be University), Karad, IND; 2 Periodontology, School of Dental Sciences, Krishna Vishwa Vidyapeeth (Deemed to be University), Karad, IND; 3 Oral Medicine and Radiology, School of Dental Sciences, Krishna Vishwa Vidyapeeth (Deemed to be University), karad, IND

**Keywords:** antibiotic prophylaxis, antibiotic prophylaxis guidelines, antimicrobial resistance, dentist, knowledge

## Abstract

Background: Antibiotic prophylaxis (AP) prescription is recommended to aid the body’s defense in clearing tissues of microbial pathogens in patients at high risk of adverse outcomes, bacteremia, or infection undergoing invasive oral and dental procedures. A variety of influences impact dentists’ decisions regarding AP prescribing. There is a paucity of data on diverse prescribing patterns of antibiotics as prophylaxis among dental students and professionals. Despite accumulating antimicrobial resistance (AMR), knowledge gaps regarding optimal prophylaxis practices persist among dental students and professionals. Considering India’s massive dental workforce, it is necessary to evaluate dentists’ current AP guideline knowledge and align local prophylaxis practices with international standards.

Objectives: This study aimed to assess the comprehension of current AP guidelines and AMR among dental students at multiple universities in India.

Materials and methods: A cross-sectional study was conducted among fourth-year students, interns, and postgraduate dental students across 18 universities in India. The study was conducted using a Google Form-based questionnaire regarding AP guidelines and AMR. The Google Form was distributed, and responses were collected. Descriptive statistics were employed, and data were presented as frequencies and percentages. Data were analyzed using IBM SPSS Statistics for Windows, version 23.0.

Results: Out of 250 dental students, 63.6% (n = 159) were female, and 36.3% (n = 91) were male. The majority were interns at 38.8% (n = 97), followed by fourth-year students at 37.6% (n = 94) and postgraduates at 23.6% (n = 59). At first glance, approximately three out of four practitioners believed that they were practicing evidence-based dentistry. However, when this self-reported familiarity was cross-referenced, only 35.1% (n = 88) actually used American Dental Association (ADA) guidelines as their primary decision-making tool, and only 26.3% (n = 66) knew the correct amoxicillin dosage one hour prior to the procedure. About 84.1% (n = 210) believed that there are almost no educational resources regarding AMR for Indian dental practitioners, whereas 89.2% (n = 223) thought that there is an urgent need to develop relevant guidelines considering AMR.

Conclusions: This study highlighted a significant knowledge-practice gap due to the absence of relevant AP guidelines considering AMR for Indian dental practitioners. The majority of dental students perceived this as one of the important catalysts for the rising rates of AMR.

## Introduction

India hosts one of the largest dental networks globally, where dentists are responsible for up to 10% of antibiotic prescribing worldwide, either as prophylaxis or therapy [1], but up to 80% of antibiotics prescribed before dental procedures to prevent infection are deemed unnecessary [2]. Antibiotic prophylaxis (AP) prior to dental procedures is indicated for immunocompromised individuals and patients with infective endocarditis (IE), metabolic disorders, or prosthetic joints. The current antibiotic prophylaxis guidelines (APGs) by the Indian Dental Association (IDA) focus on the prevention of bacterial endocarditis and the management of patients with total joint replacement [3], despite notable contradictions with guidelines from the American Dental Association (ADA) [4]. Infections caused by antimicrobial-resistant organisms kill at least 700,000 people every year and could cause 10 million deaths by the year 2050, a loss of 100 trillion US dollars to the global economy, and a 2-3.5% reduction in the world's gross domestic product if left unchecked [5]. The World Health Organization (WHO) identifies antimicrobial resistance (AMR) as a top 10 global public health threat, underscoring its role as a primary driver of modern medical crises. With this background, the present study was designed with the primary aim of appraising dental students’ knowledge and opinions regarding current APGs.

## Materials and methods

Study design

A cross-sectional survey was conducted using a convenience sampling technique among fourth-year students, dental interns, and postgraduate dental students for a period of six months (August 2025 to January 2026) across 18 universities in India. A 13-item closed-ended questionnaire (yes/no and multiple-choice) was newly developed by the authors based on established ADA and IDA APGs written in English were distributed via Google Forms (Google LLC, Mountain View, California, United States) (see Appendix for the questionnaire). Prior to full deployment, a pilot study was conducted on 15 participants to assess question clarity and comprehension; these pilot responses were excluded from the final analysis. The finalized questionnaire was distributed via Google Forms to assess knowledge and opinions regarding APGs and AMR.

Study participants

A total of 250 dental students (fourth-year students, interns, and postgraduates) were enrolled in the study. The questionnaire elicited participants' demographic details, including age, gender, year of study, university, and state, as well as their knowledge levels and clinical opinions.

Sample Size Estimation

The* *sample size was calculated using the standard sample size formula for single proportions:

$N = \frac{Z^2 \cdot P(1-P)}{E^2}$

Z = standard normal deviate at a 95% confidence level (1.96)

P = expected proportion of participants (0.50 or 50%)

E = absolute margin of error (0.07 or 7%)

Therefore,

$N = \frac{(1.96)^2 \times 0.50 \times (1 - 0.50)}{(0.07)^2} = \frac{3.8416 \times 0.25}{0.0049} = 196$

To enhance statistical power and account for potential incomplete responses, a total sample size of 250 dental students was included.

Ethical considerations

Ethical committee approval was obtained from the Institutional Review Committee of Krishna Vishwa Vidyapeeth (Deemed to be University) (Ref. No. KVV/IEC/06/2025) (Protocol Number 074/2025-2026). Informed consent was obtained from all participants prior to enrollment in the study. 

Statistical analysis

Descriptive statistics were employed to summarize the data. Categorical variables were expressed as frequencies and percentages. Data entry and statistical calculations were performed using IBM SPSS Statistics for Windows, version 23.0 (IBM Corp., Armonk, NY).

## Results

Of the 250 participating students, the majority were female (63.6%; n = 159), while 36.3% (n = 91) were male. The highest number of participating students in the study were interns (38.8%; n = 97), followed by fourth-year students (37.6%; n = 94) and postgraduates (23.6%; n = 59) (Table 1).

**Table 1:** Academic year-wise distribution of students.

Academic year	n	%
Fourth-year	94	37.6%
Interns	97	38.8%
Postgraduates	59	23.6%
Total	250	100%

Figure 1 illustrates the geographic distribution of the participating students across India, with the highest percentage from West India (35%; n = 87), followed by South India (30%; n = 75). North and East India accounted for 15% (n = 38) and 12% (n = 30), respectively, while Central India had the smallest segment at 8% (n = 20) of the total study sample.

**Figure 1:**
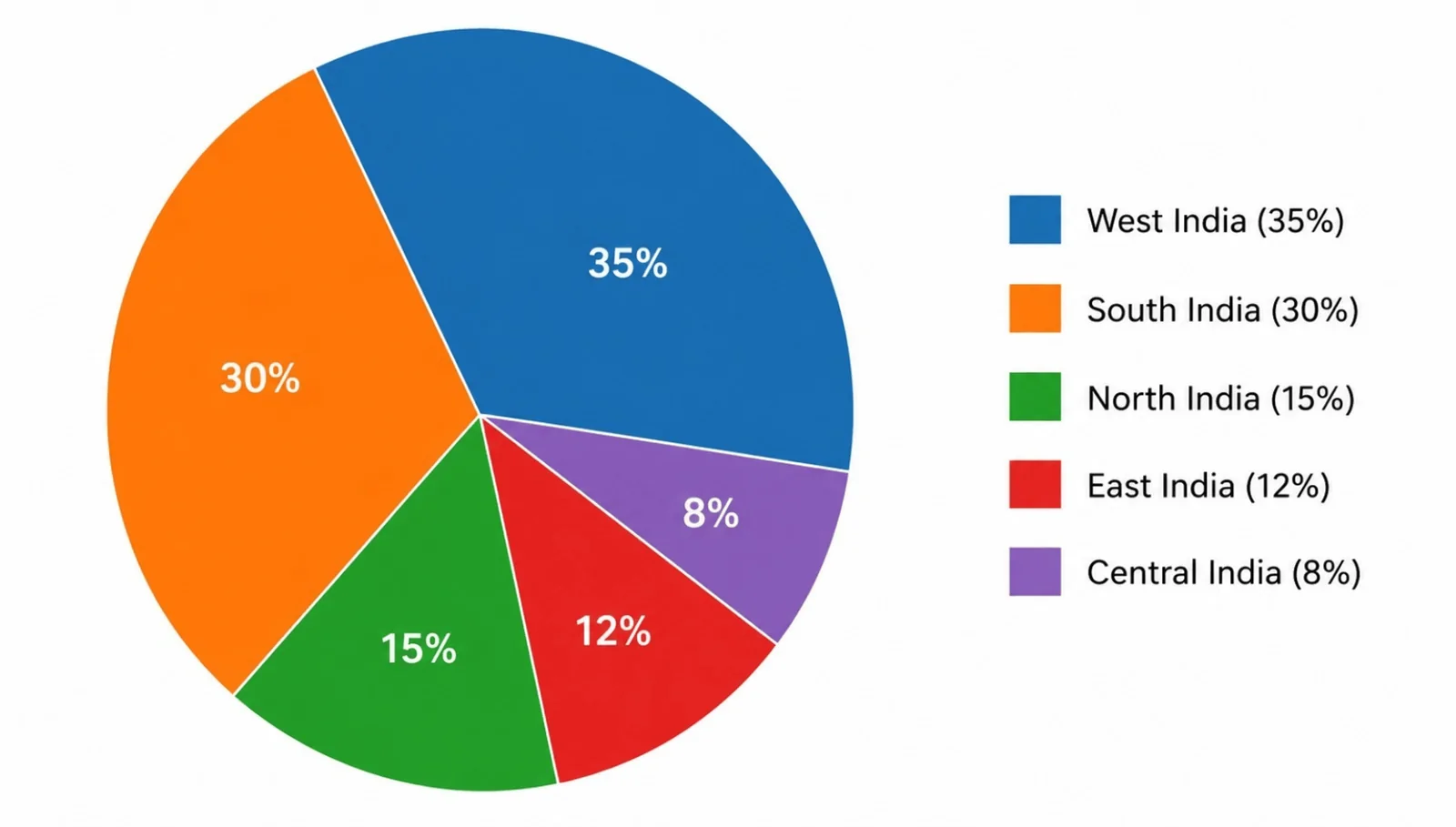
Geographic distribution of the study participants.

The age group distribution of students showed that 52.8% (n = 132) were in the age group of 21-22 years, with the least being 29 years and above at 0.8% (n = 2) (Table 2).

**Table 2:** Distribution of the study participants by age group.

Age group (in years)	n	%
21-22	132	52.8%
23-25	92	36.8%
26-28	24	9.6%
29 and above	2	0.8%
Total	250	100%

Regarding awareness of AMR, 87.6% (n = 219) of the participants responded "Yes," while 12.4% (n = 31) responded "No." Concerning current APGs, their familiarity and adherence, 74.8% (n = 187) of the dental students reported familiarity, whereas 25.2% (n = 63) reported unfamiliarity. Encouragingly, nearly three-quarters of the students perform a pre-procedure evaluation (73.2%; n = 183). However, the 26.8% (n = 67) who do not evaluate necessity before prescribing is clinically significant. The majority of students (about 55.2%; n = 138) recommended AP for all dental procedures. A majority (63.6%; n = 159) of the students prescribe a three-day course of antibiotics for irreversible pulpitis or periapical abscess prior to extraction. A significant percentage of students (64.4%; n = 161) opt for endocarditis prophylaxis during intracanal endodontic treatment. Regarding the clinical management of HIV-positive patients with a moderate CD4 count (200-500 cells/mm³), the majority of students (70.8%; n = 177) indicated that they would prescribe antibiotic prophylaxis for the removal of septic foci or abscesses, even in asymptomatic cases. Conversely, 29.2% (n = 73) of the students reported that they would not provide prophylactic coverage under these specific parameters.

In our study, 84.0% (n = 210) of the students agreed that there are almost no documented resources specifically regarding AMR or stewardship tailored for Indian dental practitioners. The majority of students (89.2%; n = 223) expressed that there is an urgent need to develop relevant APGs specifically for Indian dental practitioners, especially when considering the escalating global and national burden of AMR. The survey findings reveal an overwhelming consensus, with 93.6% (n = 234) of students believing that the absence of structured guidelines or toolkits for appropriate prophylactic prescription is a significant contributing factor to the rise of AMR in India (Table 3).

**Table 3:** Distribution of responses regarding APGs and AMR. *Denotes the guideline-recommended/evidence-based correct response according to the standard IDA guidelines. APG: antibiotic prophylaxis guideline, AMR: antimicrobial resistance, IDA: Indian Dental Association

Questions	Responses (%)	
	Yes, n(%)	No, n(%)
1) Are you aware of antimicrobial resistance (AMR)?	219 (87.6%)	31 (12.4%)
2) Are you familiar with and adhere to current antibiotic prophylaxis guidelines (APGs) for dental practitioners?	187 (74.8%)	63 (25.2%)
3) Do you evaluate whether antibiotic prophylaxis (AP) is necessary or not before any dental procedure?	183 (73.2%)*	67 (26.8%)
4) Do you recommend antibiotic prophylaxis (AP) for all dental procedures that involve manipulation of gingival tissue or the periradicular region of the tooth?	138 (55.2%)	112 (44.8%)*
5) Do you prescribe antibiotic prophylaxis (AP) of at least 3 days for every patient diagnosed with periapical abscess/ irreversible pulpitis before extraction?	159 (63.6%)	91 (36.4%)*
6) Would you prescribe endocarditis prophylactic antibiotics for a patient undergoing intracanal endodontic treatment?	161 (64.4%)	89 (35.6%)*
7) Do you prescribe antibiotic prophylaxis (AP) to an HIV positive patient with a CD4 count between 200-500 cells/mm for removal of septic foci/abscess even if asymptomatic?	177 (70.8%)*	73 (29.2%)
8) Do you believe that there are almost no educational resources regarding antimicrobial resistance (AMR) or stewardship for Indian dental practitioners in document form?	210 (84.0%)	40 (16.0%)
9) Do you think that there is an urgent need to develop relevant antibiotic prophylaxis guidelines (APGs) for Indian dental practitioners considering the burden of antimicrobial resistance (AMR)?	223 (89.2%)	27 (10.8%)
10) Do you believe that lack of guidelines or toolkit for appropriate prophylactic antibiotic prescription for Indian dentists is an important contributing factor to the rise of antimicrobial resistance (AMR) in India?	234 (93.6%)	16 (6.4%)

Regarding the decision to prescribe prophylactic antibiotics, 35.1% (n = 88) of the students were following ADA guidelines, 34.7% (n = 87) were relying on senior opinions, and 30.3% (n = 75) prescribed based on personal experience (Figure 2).

**Figure 2:**
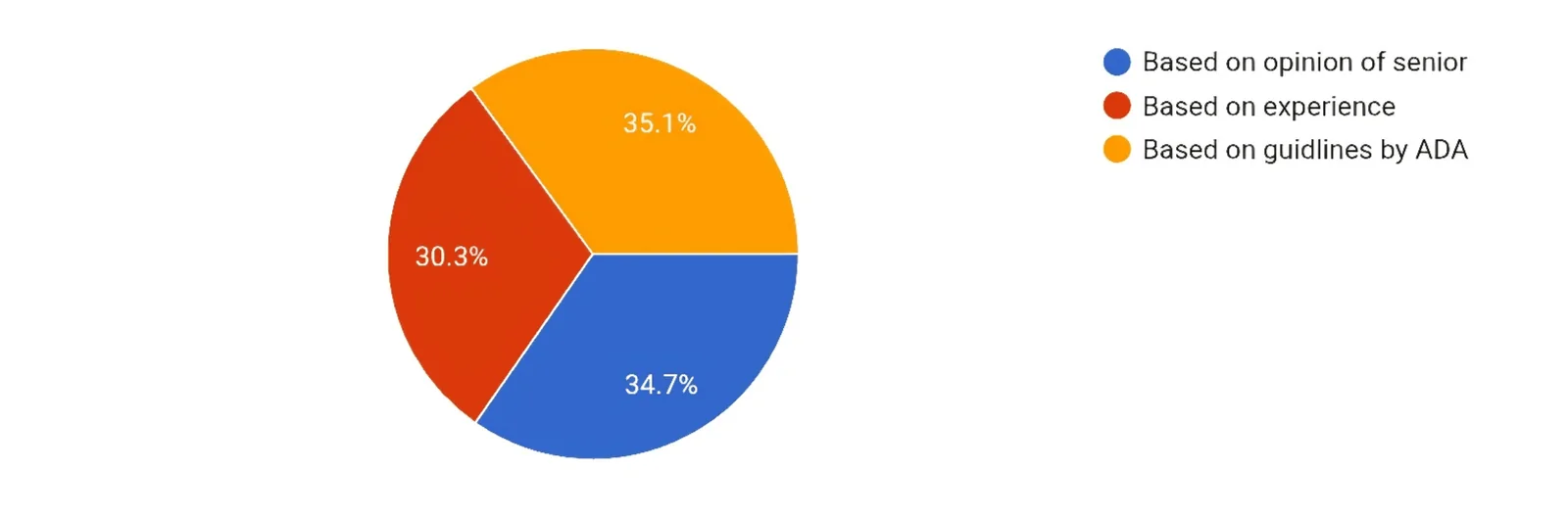
Participants' decision-making criteria for prescribing prophylactic antibiotics.

Regarding the basic mechanism of antibiotic resistance, 39.8% (n = 99) correctly identified that bacteria become resistant, while the remaining participants selected an alternative misconception. Furthermore, 22.3% (n = 56) reported that continuing dosage after symptom resolution leads to resistance, 19.1% (n = 48) noted that infection persistence despite high doses defines resistance, and 18.7% (n = 47) stated that the human body becomes resistant to antibiotics (Figure 3).

**Figure 3:**
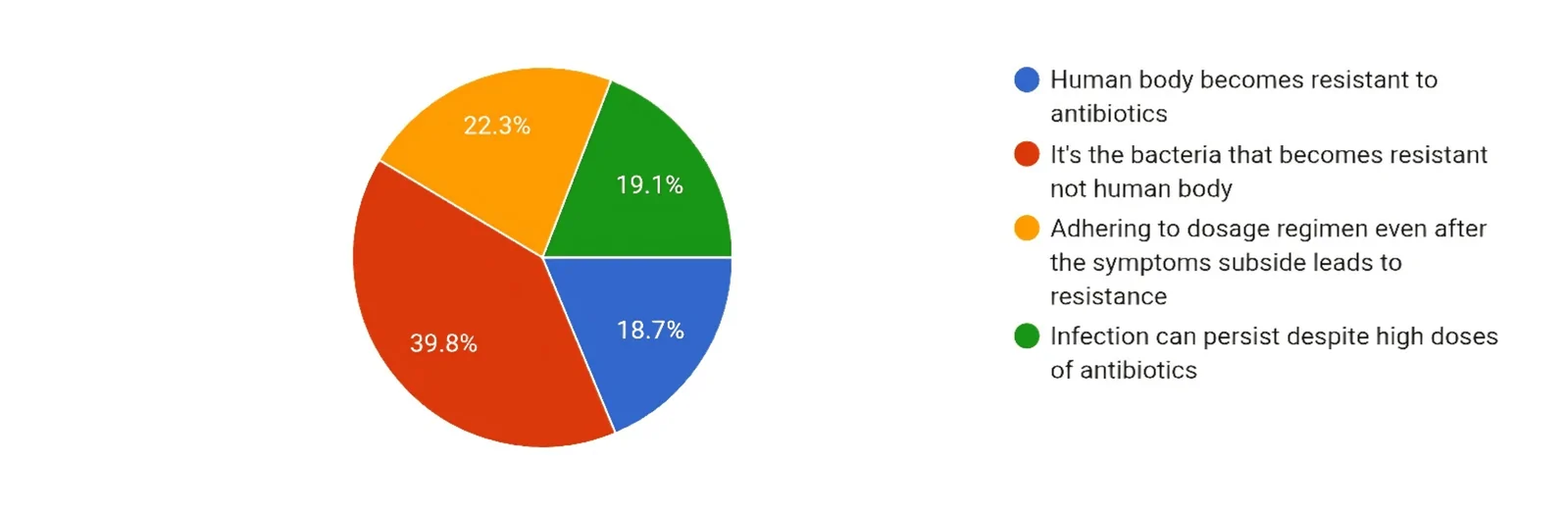
Knowledge and perceptions regarding the mechanism of antibiotic resistance.

Regarding the guideline-recommended adult prophylactic dosage of amoxicillin prior to a dental procedure (2000 mg one hour before the procedure), the students’ responses were split into four distinct groups. The largest portion (38.2%; n = 95) selected 500 mg twice daily for three days. This was closely followed by 26.7% (n = 67) who chose 500 mg twice daily for at least two days. Meanwhile, 26.3% (n = 66) opted for the 2000 mg dose given one hour before the procedure, and the remaining 8.8% (n = 22) selected the 200 mg dose one hour before the procedure (Figure 4).

**Figure 4:**
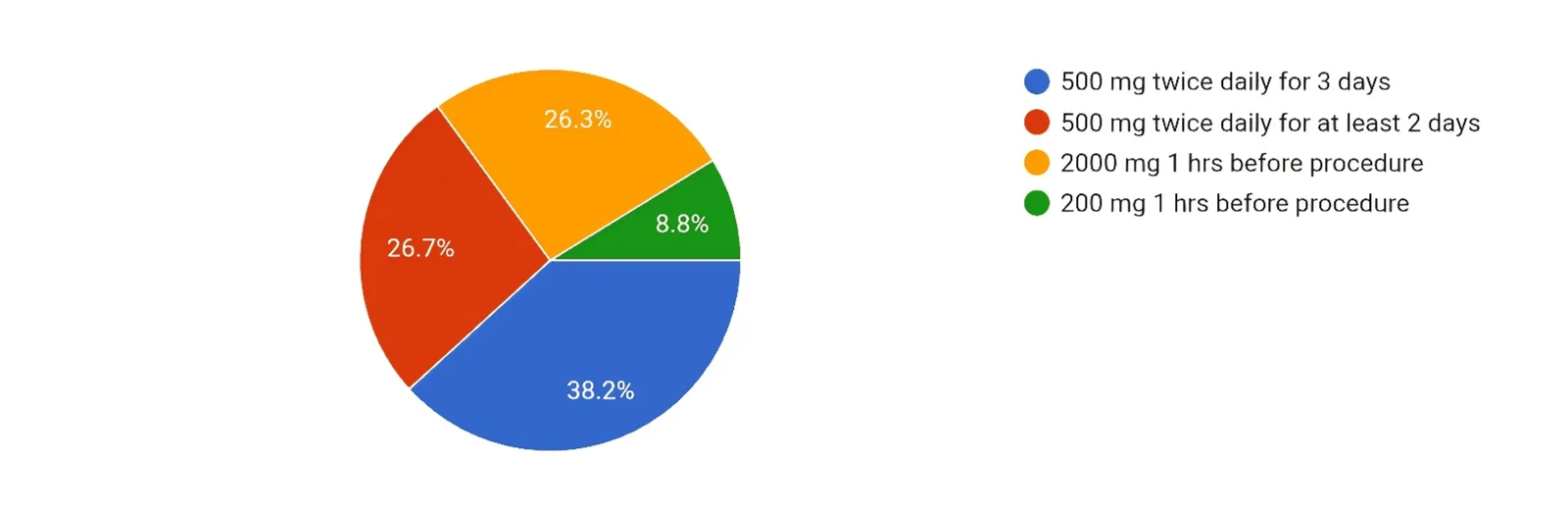
Knowledge regarding the recommended adult dosage of amoxicillin for antibiotic prophylaxis.

## Discussion

AP prevents infection during invasive dental procedures for high-risk patients. However, Indian practices lack standardized, dental-specific guidance within current Indian Council of Medical Research (ICMR) antimicrobial frameworks [6]. In addition, the Dental Council of India (DCI) undergraduate curriculum overlooks appropriate antibiotic prescribing, AMR, and stewardship, leading to a lot of confusion and contradictory knowledge and understanding among dental students [6]. This study evaluates knowledge of APGs and perception regarding optimization needs among Indian dental students, addressing a significant data gap in dental antimicrobial stewardship awareness.

The present study showed higher female predominance (63.6%) in the dental field compared to males, which is in accordance with other studies conducted globally [7-10]. The results of our study were also in accordance with a study conducted by Narang P et al. in Raipur, India [11]. Conversely, in a cross-sectional survey conducted by Puranik MP et al. in Bengaluru City, India, 54.3% were male, and 45.7% were female [12]. The majority of students were Interns (38.8%), followed by fourth-year students (37.6%) and, least, postgraduates (23.6%), which was in accordance with a study by Narang P et al. [11]. The results of our study were contradictory to a study conducted by Puranik MP et al., which showed predominance of postgraduate students (59%) [12].

The prevalence of AMR awareness in our study was 87.6%, indicating a strong foundational understanding of AMR, though it falls short of the 95.2% reported knowledge observed in studies conducted in Saudi Arabia [7]. A study conducted by Ealla KKR et al. among Dental Practitioners in Hyderabad found that while 63.8% of Indian practitioners claim to stay updated on guidelines, a massive 72.3% were completely unaware of the WHO AWaRe classification (Access, Watch, Reserve-to simplify antimicrobial stewardship) [13]. A 2026 systematic review of 14 studies by the University of Hertfordshire, involving 3,602 Indian participants, revealed that while 86% of regional research focuses on prescribing practices, only 43% specifically evaluates the actual knowledge or awareness of practitioners [14].

In this study, 74.8% of students reported being familiar with and adhering to current APGs. This reflects a trend seen in a study by Ramanarayanan V et al., where dental interns show a strong baseline of theoretical awareness, yet high prescription rates - ranging from 56% to 88% in some Indian outpatient settings - suggest a significant gap between reported knowledge and evidence-based practice [15]. Regarding the necessity of AP before any dental procedures, around 35% agreed that it is based on guidelines by the ADA. In a study conducted by Rela R et al. in the urban Indian population, APGs were followed by 67.6% of practitioners [16]. In a national survey study conducted by Šutej I et al. to evaluate dentists’ practice and compliance with current guidelines of IE prophylaxis, only 25% utilized the American Heart Association (AHA) guidelines [17].

In the present study, 73.2% of the cohort agreed on performing pre-procedure evaluations to assess the necessity of AP prior to dental procedures. The results were contradictory to a study conducted by Agarwal R et al. among dental practitioners of Chhattisgarh; 74.8% of respondents had never undergone any form of training on antibiotic prescription [18].

Only 39.8% of our cohort correctly knew that bacteria evolve resistance. This deficit aligns with international findings, including a systematic review across high-income countries reporting 20% awareness [19] and a regional study showing 5.5% accuracy among Nigerian dental students [20]. The finding that 55.4% of students endorse AP for any procedure involving gingival or periradicular manipulation reflects a potential gap in awareness or understanding of current guidelines. This inclination toward over-prescription is consistent with findings by Narang P et al. [11].

A significant challenge identified in this study is the presence of contradictory clinical information available to Indian practitioners via various digital and institutional platforms. International standards such as the ADA [4] and AHA [21] have moved toward a highly restrictive “high-risk-only” model [4]. Vague, procedural criteria overlook specific high-risk cohorts - cardiac, joint-complication, or immunosuppressed patients - contributing to substantial antibiotic overuse and improper dental prophylaxis, as Goyal R et al. demonstrated [22]. The present study revealed that 63.7% of students initiate a three-day antibiotic regimen for irreversible pulpitis or periapical abscesses prior to extraction, which was in accordance with a study by Goyal R et al. [22].

The data reveal that a significant majority (64.4%) of students opt to prescribe endocarditis prophylaxis during intracanal endodontic treatment. In a study conducted by Singh A et al. in Shimla, Himachal Pradesh, 58-67.8% of students prescribed AP [23]. Pereira WD et al. reported that 93.1% of the Indian dentists recommended AP against IE [24]. The clinical goal of prophylaxis is to achieve a high, protective concentration of the drug in the bloodstream precisely during the procedure. However, only 26.3% of students correctly selected the standard single 2000 mg pre-operative dose. The results were in accordance with IDA guidelines [3]. 

In the present study regarding the management of immunocompromised individuals, it is highly encouraging that a majority (70.8%) of the cohort indicated they would prescribe AP for an asymptomatic HIV-positive patient with a moderate CD4 count (200-500 cells/mm³) prior to the removal of a septic focus. The survey highlights a glaring deficiency in localized academic support, with 84.0% of respondents acknowledging a severe shortage of documented educational resources focused on AMR and antimicrobial stewardship specifically for Indian dental practitioners. This finding is strongly supported by recent systematic reviews within India [25].

In a direct response to this lack of resources and the escalating global threat of AMR, 89.2% of the surveyed students indicated a need for the development of targeted APGs tailored to the Indian dental setting. In a survey study conducted by Kaushik T et al. from Haryana, among medical and dental students of an Indian university, 99.1% of medical students and 95% of dental students agreed that there is a need to establish a course “Rational Use of Antibiotics” at the university level [26]. Furthermore, research into the Indian undergraduate dental curriculum has revealed that it contains minimal information on the rationale for prescribing antibiotics. The study highlights that even the ICMR treatment guidelines lack dedicated protocols for dental practitioners, forcing clinicians to rely on subjective or contradictory information [6]. The systemic nature of this knowledge gap is further evidenced by recent research indicating that the DCI undergraduate curriculum contains minimal instruction on rational prescribing and zero mention of stewardship [6].

Limitations of the study

In this study, there is a possibility of self-selection bias. Those who were already confident in their knowledge of AMR and current APGs might have been more willing to participate. Social desirability bias could be present, where respondents provide answers they believe are expected of them. Convenience sampling may limit generalizability and potential variations across different academic levels. Furthermore, the study combines fourth-year students, interns, and postgraduates into a single cohort. These are academically distinct groups with varying levels of clinical exposure and training. Their baseline knowledge is inherently different, which may obscure specific variations between the academic levels. Although data were collected from various universities across India, the findings might not be completely generalizable to all dental schools in the country due to differences in institutional protocols. Because the study relies on a self-administered questionnaire, it assesses theoretical knowledge rather than practical application. A participant might show good awareness about optimizing prophylaxis, but this study design cannot verify whether they actually implement these protocols in their daily clinical practice.

## Conclusions

This study highlights theoretical awareness regarding AMR among Indian dental students, yet reveals a critical knowledge-practice gap in the clinical application of AP. While the majority of students express familiarity with current guidelines, their reported clinical choices-demonstrated by a marked prevalence of over-prescribing for routine procedures, endodontic treatments, and non-guideline preoperative dosing-correlate with the reported reliance on senior opinions and personal experience over evidence-based protocols. These discrepancies are largely driven by the lack of dedicated training on rational antibiotic prescribing and stewardship within the undergraduate DCI curriculum. Consequently, there is an urgent need to develop and implement standardized APGs specifically formulated for the Indian dental setting. Currently, it is essential to eliminate clinical confusion and bridge the knowledge-practice gap. Considering India’s substantial number of dental professionals prescribing antibiotics is necessary to strengthen the degree to which current Indian APGs and practices for prophylactic antibiotic prescription conform to international standards and best practices. While experienced practitioners are crucial in developing and implementing guidelines, incorporating the perspective of dental students regarding guideline optimization offers several valuable benefits, such as early adoption and advocacy, as an investment in future dental care.

## Appendices

Study questionnaire

Demographic Details 

        Name

        Age

        Gender

        Year of study

        University

        State

Knowledge and Opinion Assessment

1. Are you aware of rising antimicrobial resistance (AMR)?

o Yes

o No

2. Are you familiar with and adhere to current antibiotic prophylaxis guidelines (APGs) for dental practitioners?

o Yes

o No

3. How do you approach the decision of whether to or not prescribe prophylactic antibiotics?   

o Based on the opinion of a senior

o Based on experience 

o Based on guidelines by the American Dental Association (ADA)

4. Do you evaluate whether antibiotic prophylaxis (AP) is necessary or not before any dental procedure?

o Yes

o No

5. How would you describe antibiotic resistance?

o The human body becomes resistant to antibiotics.

o It’s the bacteria that becomes resistant, not the human body.

o Adhering to the dosage regimen even after the symptoms subside leads to resistance.

o Infection can persist despite high doses of antibiotics.

6. Do you recommend antibiotic prophylaxis (AP) for all dental procedures that involve manipulation of gingival tissue or periradicular region of tooth?

o Yes

o No

7. Do you prescribe antibiotic prophylaxis (AP) of at least 3 days for every patient diagnosed with periapical abscess/ Irreversible pulpitis before extraction?

o Yes

o No

8. Would you prescribe endocarditis prophylactic antibiotics for a patient undergoing intracanal endodontic treatment?

o Yes

o No

9. According to guidelines by Indian Dental Association (IDA) recommended dosage of Amoxicillin as antibiotic prophylaxis (AP) in adults before dental procedure is _________.

o 500 mg twice daily for three days

o 500 mg twice daily for at least two days

o 2000 mg one hour before procedure

o 200 mg one hour before procedure

10. Do you prescribe antibiotic prophylaxis (AP) to an HIV-positive patient with a CD4 count of 200-500 cells/mm^3^ for the removal of septic foci/ abscess even if he is asymptomatic?

o Yes

o No

11. Do you believe that there are almost no educational resources regarding antimicrobial resistance (AMR) or stewardship for Indian dental practitioners in document form?

o Yes

o No

12. Do you think that there is an urgent need to develop relevant antibiotic prophylaxis guidelines (APGs) for Indian dental practitioners considering the burden of antimicrobial resistance (AMR)?

o Yes

o No

13. Do you believe that the lack of guidelines for appropriate prophylactic antibiotic prescription for Indian dentists is an important contributing factor to the rise of antimicrobial resistance (AMR) in India?

o Yes

o No
